# Supplementary material for: The role of 5-HTTLPR in autism spectrum disorder: New evidence and a meta-analysis of this polymorphism in Latin American population with psychiatric disorders
Source: PLoS One. 2020 Jul 2;15(7):e0235512. doi: 10.1371/journal.pone.0235512 (PMC7332001; doi:10.1371/journal.pone.0235512)
Supplement: S1 Table — (DOCX) [file pone.0235512.s001.docx]

**S1 Table. Genotyping data for each trio and ADOS score for each individual with idiopathic ASD.**

| Participant ID | 5-HTTLPR Genotype | | | Gender | ADOS score for individual with idiopathic ASD | | |
| --- | --- | --- | --- | --- | --- | --- | --- |
|  | Father | Mother | Individual with idiopathic ASD |  | Social communication | Restricted/Repetitive behaviors | Social interaction |
| ASD_2 | SL | SL | SL | Male | 8 | 4 | 12 |
| ASD_3 | SS | SL | SS | Male | 3 | 1 | 9 |
| ASD_5 | LL | LL | LL | Male | 5 | 6 | 9 |
| ASD_8 | SS | SS | SS | Male | 6 | 6 | 13 |
| ASD_13 | SL | LL | LL | Male | 6 | 2 | 10 |
| ASD_14 | LL | SS | SL | Female | 4 | 3 | 7 |
| ASD_21 | SL | SL | SL | Male | 4 | 1 | 7 |
| ASD_23 | SL | SS | SS | Male | 8 | 4 | 10 |
| ASD_24 | SL | SS | SL | Male |  |  |  |
| ASD_25 | SL | SL | SS | Male | 7 | 2 | 7 |
| ASD_26 | SL | SS | SL | Male | 8 | 1 | 13 |
| ASD_27 | SS | SS | SS | Male | 5 | 1 | 9 |
| ASD_30 | SL | LL | SL | Female | 4 | 6 | 5 |
| ASD_32 | LL | SL | LL | Male | 6 | 7 | 9 |
| ASD_33 | SS | LL | SL | Male | 3 | 2 | 9 |
| ASD_34 | SS | SL | SS | Male | 3 | 2 | 6 |
| ASD_36 | LL | SS | SL | Male | 5 | 1 | 9 |
| ASD_37 | SS | SL | SL | Male | 9 | 3 | 12 |
| ASD_39 | SS | SL | SL | Male | 7 | 0 | 12 |
| ASD_40 | SS | LL | SL | Male | 6 | 7 | 9 |
| ASD_41 | SS | SS | SS | Male | 8 | 3 | 8 |
| ASD_48 | SL | LL | LL | Male | 3 | 2 | 4 |
| ASD_50 | SL | SS | SS | Male | 3 | 0 | 8 |
| ASD_51 | SS | SL | SL | Female | 4 | 3 | 8 |
| ASD_53 | SL | SL | LL | Male | 2 | 2 | 13 |
| ASD_54 | SS | SS | SS | Male |  |  |  |
| ASD_55 | LL | SS | SL | Male | 5 | 3 | 6 |
| ASD_56 | SS | SL | SS | Female | 3 | 2 | 4 |
| ASD_57 | SS | LL | SL | Male | 6 | 5 | 11 |
| ASD_60 | SL | SL | SS | Male | 3 | 4 | 9 |
| ASD_63 | SL | SS | SL | Male | 4 | 4 | 9 |
| ASD_64 | SL | SS | SL | Male | 5 | 1 | 9 |
| ASD_65 | SL | LL | SL | Male | 8 | 6 | 14 |
| ASD_66 | SS | SL | SL | Male | 7 | 4 | 8 |
| ASD_67 | SS | SS | SS | Male | 6 | 3 | 13 |
| ASD_68 | SS | SL | SL | Male | 7 | 2 | 11 |
| ASD_69 | SL | SL | SL | Male | 5 | 3 | 14 |
| ASD_71 | SL | SL | SS | Female | 5 | 3 | 5 |
| ASD_73 | SS | SL | SS | Male | 6 | 6 | 11 |
| ASD_74 | LL | SL | SL | Male | 8 | 4 | 10 |
| ASD_75 | LL | LL | LL | Male | 5 | 3 | 9 |
| ASD_76 | SL | SL | LL | Male | 6 | 3 | 11 |
| ASD_77 | SS | SS | SS | Male | 9 | 2 | 11 |
| ASD_78 | SL | SS | SS | Male | 9 | 4 | 13 |
| ASD_80 | SL | LL | SL | Male | 6 | 2 | 9 |
| ASD_81 | SL | SL | SS | Male | 5 | 2 | 10 |
| ASD_82 | SS | SS | SS | Male | 6 | 3 | 11 |
| ASD_83 | LL | LL | LL | Male | 4 | 3 | 7 |
| ASD_84 | SS | LL | SL | Male | 6 | 3 | 2 |
| ASD_85 | SS | SS | SS | Male | 2 | 3 | 4 |
| ASD_87 | SL | LL | LL | Female | 5 | 2 | 8 |
| ASD_88 | LL | LL | LL | Male | 5 | 2 | 6 |
| ASD_90 | LL | SL | LL | Male | 4 | 4 | 10 |
| ASD_91 | SL | LL | LL | Male | 4 | 2 | 7 |
| ASD_92 | SL | LL | LL | Female | 7 | 2 | 5 |
| ASD_96 | SS | LL | SL | Male | 6 | 4 | 7 |
| ASD_97 | SL | SS | SS | Male | 4 | 1 | 3 |
| ASD_98 | SL | SL | SL | Female | 9 | 1 | 9 |
| ASD_99 | SL | SL | LL | Male | 4 | 4 | 10 |
| ASD_100 | SS | SL | SS | Male | 7 | 4 | 11 |
| ASD_101 | LL | SL | LL | Male | 9 | 4 | 11 |
| ASD_102 | SS | SL | SS | Male | 4 | 4 | 7 |
| ASD_103 | SS | SL | SL | Female | 4 | 1 | 3 |
| ASD_104 | SL | SS | SS | Male | 9 | 1 | 9 |
| ASD_105 | SL | SS | SL | Female | 7 | 2 | 13 |
| ASD_106 | SL | SL | SL | Male | 7 | 4 | 11 |
| ASD_108 | SL | SL | LL | Male | 7 | 4 | 7 |
| ASD_110 | SL | SS | SS | Male | 5 | 4 | 5 |
| ASD_111 | SL | SS | SS | Male |  |  |  |
| ASD_112 | SL | SS | SS | Male | 6 | 2 | 12 |
| ASD_114 | LL | SS | SL | Male | 8 | 3 | 10 |
| ASD_115 | LL | SS | SL | Male | 8 | 3 | 9 |
| ASD_116 | LL | SL | LL | Male | 7 | 4 | 7 |
| ASD_118 | SL | SS | SL | Male | 7 | 3 | 10 |
| ASD_119 | LL | SL | LL | Female | 3 | 2 | 6 |
| ASD_120 | LL | SL | SL | Female | 3 | 3 | 5 |
| ASD_121 | SS | LL | SL | Female | 3 | 2 | 7 |
| ASD_123 | SL | SL | SL | Male | 5 | 3 | 8 |
| ASD_126 | SS | SS | SS | Male | 5 | 2 | 5 |
| ASD_129 | SS | SL | SS | Male | 6 | 2 | 7 |
| ASD_130 | SS | SS | SS | Male | 3 | 2 | 4 |
| ASD_131 | SS | SL | SS | Male | 3 | 2 | 7 |
| ASD_133 | SL | SS | SS | Female | 8 | 6 | 9 |
| ASD_134 | SS | SS | SS | Male | 6 | 3 | 10 |
| ASD_140 | LL | SL | SL | Male | 6 | 2 | 7 |
| ASD_141 | SL | SL | LL | Male | 4 | 0 | 8 |
| ASD_142 | SL | SS | SS | Male | 4 | 0 | 3 |
| ASD_145 | SL | SS | SL | Male | 6 | 2 | 8 |
| ASD_146 | SL | LL | LL | Male | 6 | 0 | 8 |
| ASD_147 | SL | SL | LL | Male | 7 | 5 | 10 |
| ASD_148 | SL | SS | SL | Male | 7 | 2 | 9 |
| ASD_149 | SS | SS | SS | Male | 6 | 3 | 10 |
| ASD_150 | LL | SS | SL | Male | 4 | 0 | 3 |
| ASD_151 | SS | SS | SS | Male | 8 | 6 | 10 |
| ASD_152 | SS | LL | SL | Male | 6 | 3 | 9 |
| ASD_153 | SS | SL | SS | Male | 8 | 4 | 11 |
| ASD_154 | LL | LL | LL | Male | 7 | 2 | 9 |
| ASD_155 | SL | LL | SL | Female | 6 | 3 | 10 |
| ASD_156 | SS | SL | SS | Male | 7 | 6 | 9 |
| ASD_157 | SL | LL | SL | Male | 5 | 3 | 6 |
| ASD_159 | SS | SL | SL | Male | 6 | 3 | 9 |
| ASD_160 | SL | SS | SS | Male | 8 | 4 | 11 |
| ASD_161 | SL | SL | SS | Male |  |  |  |
| ASD_162 | SL | SS | SS | Male | 4 | 2 | 9 |
| ASD_163 | SL | SS | SS | Male | 5 | 1 | 8 |
