# Supplementary material for: The role of 5-HTTLPR in autism spectrum disorder: New evidence and a meta-analysis of this polymorphism in Latin American population with psychiatric disorders
Source: PLoS One. 2020 Jul 2;15(7):e0235512. doi: 10.1371/journal.pone.0235512 (PMC7332001; doi:10.1371/journal.pone.0235512)
Supplement: S2 Table — (DOCX) [file pone.0235512.s002.docx]

**S2 Table. Genotyping data for each unrelated control.**

| Control ID | Sex | 5-HTTLPR Genotype |
| --- | --- | --- |
| CC01 | Male | SL |
| CC02 | Male | SS |
| CC03 | Female | SS |
| CC04 | Male | SL |
| CC05 | Female | LL |
| CC06 | Female | SL |
| CC07 | Female | SL |
| CC08 | Female | SL |
| CC09 | Male | LL |
| CC10 | Female | SL |
| CC12 | Female | LL |
| CC13 | Female | LL |
| CC14 | Female | SL |
| CC15 | Female | LL |
| CC17 | Female | SL |
| CC20 | Female | SL |
| CC21 | Female | LL |
| CC25 | Female | SL |
| CC31 | Female | LL |
| CC33 | Male | SL |
| CC34 | Female | SL |
| CC35 | Female | SL |
| CC36 | Female | SS |
| CC37 | Female | SS |
| CC39 | Female | SS |
| CC40 | Female | SL |
| CC41 | Female | SL |
| CI01 | Male | SS |
| CI02 | Male | SS |
| CI03 | Female | SS |
| CI04 | Female | SS |
| CI05 | Female | SS |
| CI06 | Female | LL |
| CI07 | Female | SL |
| CI08 | Female | SL |
| CI09 | Female | SL |
| CI10 | Female | SL |
| CI11 | Female | SL |
| CI12 | Female | SL |
| CI13 | Female | LL |
| CI14 | Female | SL |
| CI15 | Female | LL |
| CI16 | Female | LL |
| CI17 | Female | LL |
| CI18 | Male | SS |
| CI19 | Male | SL |
| CI20 | Male | LL |
| CI21 | Male | SL |
| CM01 | Female | LL |
| CM02 | Female | SL |
| CM03 | Female | LL |
| CM04 | Female | SL |
| CM05 | Female | SL |
| CM06 | Female | SL |
| CM07 | Female | SL |
| CM08 | Female | SL |
| CM09 | Female | LL |
| CM10 | Male | SS |
| CM11 | Male | SL |
| CM12 | Male | SS |
| CM13 | Male | SL |
| CM14 | Male | SL |
| CM15 | Female | LL |
| CM16 | Male | SL |
| CU02 | Female | SL |
| CU03 | Female | SL |
| CU04 | Male | SS |
| CU05 | Female | SS |
| CU06 | Female | SL |
| CU08 | Female | SS |
| CU09 | Female | SL |
| CU10 | Female | SL |
| CU11 | Female | SL |
| CU13 | Female | LL |
| CU14 | Male | SL |
| CU16 | Female | SL |
| CU17 | Female | SL |
| CU19 | Female | SL |
| CU20 | Female | SS |
| CU21 | Female | LL |
| CU23 | Male | SL |
| CU24 | Male | SS |
| CU25 | Female | LL |
| CU26 | Female | SL |
| CU27 | Female | SL |
| CU29 | Male | SL |
| CU30 | Male | SL |
| CU31 | Male | SS |
| CU32 | Female | LL |
| L01 | Female | SS |
| L02 | Female | LL |
| L03 | Male | LL |
| L04 | Male | SS |
| L05 | Female | SS |
| L06 | Female | LL |
| L07 | Female | SS |
| L08 | Male | SL |
| L09 | Female | SL |
| L10 | Female | SL |
| L12 | Female | SS |
| L13 | Female | SS |
| L14 | Female | SL |
| L15 | Female | SL |
| L16 | Female | SL |
| L17 | Female | SS |
| L19 | Female | LL |
| L20 | Male | LL |
| L21 | Female | SS |
| L22 | Female | SL |
| L23 | Female | SL |
| L24 | Female | SS |
| L25 | Female | SL |
| L26 | Female | SL |
| L27 | Female | SL |
| L28 | Female | SL |
| L29 | Female | SS |
| L31 | Female | SL |
| L32 | Female | SL |
| L33 | Female | SL |
| L34 | Female | SS |
| L35 | Female | SL |
| L36 | Male | SL |
| L37 | Male | SL |
| L38 | Female | SL |
| L39 | Female | LL |
| L40 | Female | SS |
| L41 | Female | SL |
| L42 | Female | SS |
| L43 | Female | LL |
| L44 | Female | LL |
| L45 | Female | SS |
| L46 | Female | LL |
| L48 | Female | SL |
| L49 | Female | SL |
| L50 | Female | SL |
| L51 | Female | LL |
| L52 | Female | LL |
| L53 | Male | SS |
| L54 | Female | SL |
| L55 | Female | SL |
| L58 | Female | LL |
| L59 | Female | SS |
| L60 | Female | SL |
| L61 | Female | SS |
| L62 | Female | SL |
| L63 | Female | SL |
| L64 | Female | SL |
| L65 | Male | SL |
| L66 | Female | SS |
| L67 | Female | SL |
| L68 | Female | SS |
| L69 | Female | SS |
| L70 | Female | SL |
| L71 | Female | SS |
| L72 | Female | LL |
| L73 | Male | LL |
| L75 | Female | SS |
| L76 | Female | SL |
| L77 | Female | LL |
| L78 | Female | SS |
| L79 | Female | LL |
| L80 | Female | SL |
| L81 | Female | SL |
| L82 | Female | SL |
| L83 | Female | SL |
| L84 | Female | SS |
| L87 | Male | SL |
| L88 | Male | SL |
| L90 | Female | SL |
| L91 | Female | SS |
| L94 | Male | LL |
