# Supplementary material for: The role of 5-HTTLPR in autism spectrum disorder: New evidence and a meta-analysis of this polymorphism in Latin American population with psychiatric disorders
Source: PLoS One. 2020 Jul 2;15(7):e0235512. doi: 10.1371/journal.pone.0235512 (PMC7332001; doi:10.1371/journal.pone.0235512)
Supplement: S3 Table — (DOCX) [file pone.0235512.s003.docx]

**S3 Table. Results of case-control association test between ASD and 5-HTTLPR polymorphism**

| Genetic model | **AFFECTED** | **UNAFFECTED** | ***p*** |
| --- | --- | --- | --- |
| LL/LS/SS | 22/42/41 | 38/89/44 | 0.0562 |
| L vs S | 86/124 | 165/177 | 0.113 |
| LL/LS vs SS | 64/41 | 127/44 | **0.02277** |
| LL vs SL/SS | 22/83 | 38/133 | 0.8809 |
