# Supplementary material for: The role of 5-HTTLPR in autism spectrum disorder: New evidence and a meta-analysis of this polymorphism in Latin American population with psychiatric disorders
Source: PLoS One. 2020 Jul 2;15(7):e0235512. doi: 10.1371/journal.pone.0235512 (PMC7332001; doi:10.1371/journal.pone.0235512)
Supplement: S4 Table — TDT was performed with families having heterozygotes parents (49 fathers and 42 mothers). (DOCX) [file pone.0235512.s004.docx]

**S4 Table. Transmission Disequilibrium Test in Colombian trios with ASD.**

|  | Transmitted | Not transmitted |
| --- | --- | --- |
| **Paternal** |  |  |
| Short | 26 | 23 |
| Long | 23 | 26 |
| **Maternal** |  |  |
| Short | 21 | 21 |
| Long | 21 | 21 |
| ***TDT*** X ^2^=0,0989 *p*=0,7532 | | |

TDT was performed with families having heterozygotes parents (49 fathers and 42 mothers).
