# Supplementary material for: The role of 5-HTTLPR in autism spectrum disorder: New evidence and a meta-analysis of this polymorphism in Latin American population with psychiatric disorders
Source: PLoS One. 2020 Jul 2;15(7):e0235512. doi: 10.1371/journal.pone.0235512 (PMC7332001; doi:10.1371/journal.pone.0235512)
Supplement: S5 Table — Two sub-categories were well-thought-out, patients who loss the acquired skills at 2 years old were classified as “ASD with regressive development” and patients with ASD and higher cognitive skills were classified as “High functioning ASD”. Intellectual disability was evaluated during clinical consultation without standardized test. Aggressive behaviors were analyzed according to ADIR reports. (DOCX) [file pone.0235512.s005.docx]

**S5 Table. Phenotypic traits in individuals with ASD according to 5-HTTLPR genotypes.**

| **Genotype** | **ASD subtype** | | | | **Comorbidities** | | | |
| --- | --- | --- | --- | --- | --- | --- | --- | --- |
|  | **ASD** | **High functioning** | | **ASD with regressive development** | **Epilepsy** | **Intellectual disability** | **Self-Agressive Behaviors** | **Agressive behaviors with othersd** |
| **SS** | 28 | 8 | | 5 | 3 | 10 | 15 | 4 |
| **SL** | 31 | 5 | | 6 | 3 | 11 | 8 | 12 |
| **LL** | 16 | 2 | | 4 | 2 | 7 | 6 | 19 |
| **Total of individuals** | 75 | 15 | 15 | | 8 | 18 | 29 | 35 |

Two sub-categories were well-thought-out, patients who loss the acquired skills at 2 years old were classified as “ASD with regressive development” and patients with ASD and higher cognitive skills were classified as “High functioning ASD”. Intellectual disability was evaluated during clinical consultation without standardized test. Aggressive behaviors were analyzed according to ADIR reports.
