# Supplementary material for: The role of 5-HTTLPR in autism spectrum disorder: New evidence and a meta-analysis of this polymorphism in Latin American population with psychiatric disorders
Source: PLoS One. 2020 Jul 2;15(7):e0235512. doi: 10.1371/journal.pone.0235512 (PMC7332001; doi:10.1371/journal.pone.0235512)
Supplement: S6 Table — (DOCX) [file pone.0235512.s006.docx]

**S6 Table. Sensitivity analysis**

| Ommited Study | S vs L | | | | SS vs SL+LL | | | | LL vs SL+SS | | | |
| --- | --- | --- | --- | --- | --- | --- | --- | --- | --- | --- | --- | --- |
|  | OR | p | I^2^ | p | OR | p | I^2^ | p | OR | p | I^2^ | p |
| Present | 1.0570 [0.9741; 1.1470] | 0.1836 | 32.9% | 0.0728 | 1.0791 [0.9411; 1.2372] | 0.2754 | 19.4% | 0.2089 | 0.9255 [0.8140; 1.0523] | 0.2372 | 15.2% | 0.2610 |
| (Ospina-Duque et al., 2000) | 1.0994 [1.0126; 1.1936] | 0.0239 | 19.4% | 0.2083 | 1.1158 [0.9744; 1.2777] | 0.1130 | 20.8% | 0.1918 | 0.9031 [0.7943; 1.0268] | 0.1196 | 14.7% | 0.2680 |
| (Pérez-Olmos, Bustamante, & Ibáñez-Pinilla, 2016) | 1.0665 [0.9838; 1.1560] | 0.1179 | 35.9% | 0.0527 | 1.0985 [0.9607; 1.2562] | 0.1696 | 23.2% | 0.1642 | 0.9180 [0.8081; 1.0429] | 0.1887 | 17.4% | 0.2327 |
| (Ramos, Arias, Salazar, Vélez, & Pardo, 2012) | 1.0838 [0.9989; 1.1760] | 0.0533 | 32.2.% | 0.0533 | 1.1250 [0.9813; 1.2897] | 0.0913 | 18.5% | 0.2199 | 0.9025 [0.7938; 1.0260] | 0.1168 | 14.2% | 0.2746 |
| (Escobar, Calderón, & Moreno, 2011) | 1.0617 [0.9796; 1.1507] | 0.1447 | 33.3% | 0.0700 | 1.0881 [0.9516; 1.2440] | 0.2169 | 19.7% | 0.2046 | 0.9242 [0.8138; 1.0495] | 0.2245 | 14% | 0.2764 |
| (Camarena et al., 2001) | 1.0599 [0.9768; 1.1500] | 0.1625 | 34.2% | 0.0635 | 1.0853 [0.9468; 1.2441] | 0.2397 | 21.3% | 0.1860 | 0.9244 [0.8131; 1.0509] | 0.2295 | 15.6% | 0.2558 |
| (Peralta-Leal et al., 2012). | 1.0994 [1.0126; 1.1936] | 0.0239 | 19.4% | 0.2083 | 1.1510 [1.0027; 1.3212] | 0.0457 | 5,7% | 0.3846 | 0.8887 [0.7811; 1.0112] | 0.0733 | 2.1% | 0.4316 |
| (Durán-González et al., 2018) | 1.0866 [1.0025; 1.1779] | 0.0434 | 23.4% | 0.1622 | 1.1251 [0.9838; 1.2866] | 0.0851 | 10.5% | 0.3216 | 0.8970 [0.7897; 1.0189] | 0.0947 | 5% | 0.3941 |
| (Sarmiento-Hernandez_et_al.,_2019) | 1.0290 [0.9468; 1.1184] | 0.5002 | 8..3% | 0.3513 | 1.0416 [0.9050; 1.1987] | 0.5699 | 3.8% | 0.4102 | 0.9579 [0.8407; 1.0913] | 0.5175 | 0% | 0.6002 |
| (Cajal et al., 2012) | 1.0658 [0.9827; 1.1560] | 0.1240 | 35.9% | 0.0526 | 1.0952 [0.9567; 1.2538] | 0.1873 | 23% | 0.1671 | 0.9188 [0.8082; 1.0445] | 0.1955 | 17.4% | 0.2330 |
| (Neves et al., 2008) | 1.0708 [0.9862; 1.1627] | 0.1032 | 36.3% | 0.0502 | 1.1026 [0.9629; 1.2625] | 0.1577 | 23.3.% | 0.1636 | 0.9114 [0.7991; 1.0395] | 0.1667 | 17.8% | 0.2288 |
| (Mendes De Oliveira et al., 1998) | 1.0656 [0.9822; 1.1560] | 0.1265 | 39% | 0.0388 | 1.0965 [0.9550; 1.2589] | 0.1913 | 30.5% | 0.1021 | 0.9160 [0.7997; 1.0492] | 0.2054 | 25.5% | 0.1495 |
| (Oliveira et al., 2000) | 1.0629 [0.9768; 1.1566] | 0.1570 | 41.5% | 0.0306 | 1.0913 [0.9501; 1.2534] | 0.2165 | 29.6% | 0.1105 | 0.9249 [0.8078; 1.0588] | 0.2578 | 23.7% | 0.1692 |
| (Bortoluzzi et al., 2014) | 1.0736 [0.9897; 1.1646] | 0.0870 | 36% | 0.0521 | 1.1054 [0.9660; 1.2650] | 0.1452 | 23% | 0.1665 | 0.9098 [0.7998; 1.0350] | 0.1509 | 17.4% | 0.2328 |
| (Segal, Pujol, Birck, Gus Manfro, & Leistner-Segal, 2006) | 1.0649 [0.9817; 1.1552] | 0.1295 | 35.7% | 0.0536 | 1.0953 [0.9569; 1.2537] | 0.1868 | 22.9% | 0.1673 | 0.9196 [0.8086; 1.0459] | 0.2018 | 17.3% | 0.2342 |
| (Longo, Schüler-Faccini, Brandalize, dos Santos Riesgo, & Bau, 2009) | 1.0689 [0.9844; 1.1606] | 0.1128 | 36.3% | 0.0502 | 1.0709 [0.9341; 1.2279] | 0.3259 | 15.4% | 0.2578 | 0.8884 [0.7798; 1.0122] | 0.0754 | 7.6% | 0.3606 |
| (Schenkel et al., 2011) | 1.0779 [0.9927; 1.1704] | 0.0741 | 35.3% | 0.0562 | 1.1375 [0.9925; 1.3038] | 0.0641 | 10.6% | 0.3206 | 0.9350 [0.8207; 1.0653] | 0.3127 | 12.2% | 0.3001 |
| (Krelling et al., 2008) | 1.0813 [0.9974; 1.1723] | 0.0577 | 31.4% | 0.0845 | 1.1144 [0.9746; 1.2742] | 0.1133 | 19.4% | 0.2084 | 0.9002 [0.7921; 1.0231] | 0.1074 | 11.7% | 0.3065 |
| (Meira-Lima et al., 2004) | 1.0694 [0.9858; 1.1601] | 0.1061 | 36.3% | 0.0502 | 1.0999 [0.9601; 1.2600] | 0.1698 | 23.3% | 0.1632 | 0.9145 [0.8044; 1.0396] | 0.1718 | 17.8% | 0.2276 |
