# Supplementary material for: The role of 5-HTTLPR in autism spectrum disorder: New evidence and a meta-analysis of this polymorphism in Latin American population with psychiatric disorders
Source: PLoS One. 2020 Jul 2;15(7):e0235512. doi: 10.1371/journal.pone.0235512 (PMC7332001; doi:10.1371/journal.pone.0235512)
Supplement: S7 Table — This table summarizes some results of studies evaluating severity of ASD symptoms and the serotoninergic system. (DOCX) [file pone.0235512.s007.docx]

**S7 Table. 5-HTTLPR polymorphism and ASD severity.**

| **Population** | **Sample size** | **Scale to evaluate ASD symptoms** | **Serotonin system evaluated** | **Result** | **Reference** |
| --- | --- | --- | --- | --- | --- |
| France | 71 | ADI-R | Serotonin polymorphism | S allele associated with more severe impairments in the social and communication domains | Tordjman S et al 2001 |
| Brazil | 151 | CARS | Serotonin polymorphism | No association | Longo et al. 2009 |
| Indian | 169 | CARS | Serotonin polymorphism and serotonin level in blood | No association | Jaiswal et al 2015 |
| Mix | 73 | ADIR / ADOS | Serotonin polymorphism | SS/SL genotype related with failure to use nonverbal communication to regulate social interaction and LL with stereotyped and repetitive motor mannerisms | Brune C et al 2006 |
| USA | 78 | ADIR | Serotonin level in blood | No association with core ASD symptoms, but inverse relation between serotonin level and self-injury | Kolevzon A et al 2010 |
| USA | 118 | ADIR / ADOS | Serotonin polymorphism | LA/LA genotype exhibited more severe symptoms ASD social deficits | Gadow KD et al 2013 |

This table summarizes some results of studies evaluating severity of ASD symptoms and the serotoninergic system.
