# Supplementary material for: The role of 5-HTTLPR in autism spectrum disorder: New evidence and a meta-analysis of this polymorphism in Latin American population with psychiatric disorders
Source: PLoS One. 2020 Jul 2;15(7):e0235512. doi: 10.1371/journal.pone.0235512 (PMC7332001; doi:10.1371/journal.pone.0235512)
Supplement: S1 Fig — (DOCX) [file pone.0235512.s008.docx]

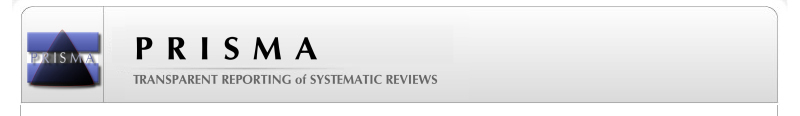
**PRISMA 2009 Flow Diagram**

**Scielo Database**

(httlpr OR SLC6A4 OR HTT) AND (Autism OR Depressive OR Depression OR Bipolar OR Epilepsy OR Schizophrenia OR Anxiety)

(n=20)

**PubMed Database**

“httlpr” “SLC6A4” AND (colombia OR argentina OR chile OR brazil OR ecuador OR peru OR mexico OR venezuela OR bolivia OR paraguay OR uruguay OR guyana OR "Costa Rica" OR guatemala OR salvador OR nicaragua OR honduras)

Filter: FullText

(n=52)

**ScienceDirect Database**

“httlpr” “SLC6A4” AND (colombia OR argentina OR chile OR brazil OR ecuador OR peru OR mexico OR venezuela OR bolivia OR paraguay OR uruguay OR guyana OR "Costa Rica" OR guatemala OR salvador OR nicaragua OR honduras)

Filter: Research Article

(n=40)

## Identification

## Included

## Eligibility

## Screening

Records identified through databases searching
(n =112)

Records after duplicates removed
(n = 98 )

Records excluded

(n = 63)*.

Records screened by title and abstract
(n = 98)

Full-text articles assessed for eligibility
(n = 35)

Studies included in qualitative synthesis
(n = 18 )

Studies included in quantitative synthesis (meta-analysis)
(n =18)

Full-text articles excluded (n = 17)^#^.

**S1 Fig PRISMA flow diagram.** *Records screened by title and abstract were removed because they did not evaluate psychiatric disorders, no genotype was present for the 5-HTTLPR polymorphism or because they corresponded to reviews. ^#^Full text articles assessed were excluded because they were family-based studies, studies evaluating cases and no controls or population without a clinical diagnosis, studies that did not evaluate the Latin American population or the association of 5-HTTLPR with psychiatric disorders and studies with insufficient available data to calculate the allelic/genotypic frequencies. If overlapped samples were used in different studies, we kept the study with the largest sample size. The 18 included studies had a publication’s date between 1998 – 2018.
