# Supplementary figures and images for: The role of 5-HTTLPR in autism spectrum disorder: New evidence and a meta-analysis of this polymorphism in Latin American population with psychiatric disorders
Source: PLoS One. 2020 Jul 2;15(7):e0235512. doi: 10.1371/journal.pone.0235512 (PMC7332001; doi:10.1371/journal.pone.0235512)

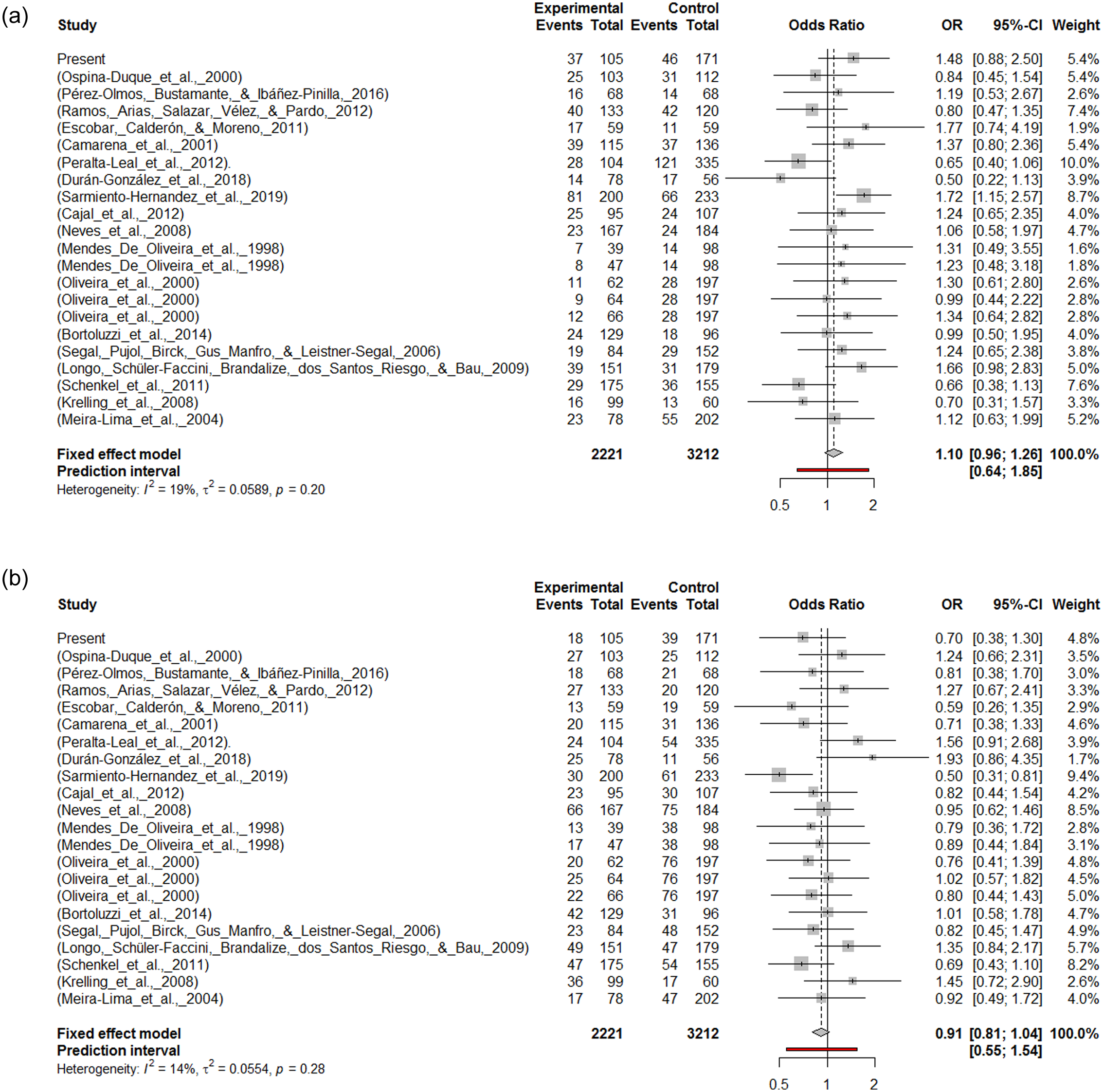

Supplement: S2 Fig — The trait and country for each study and are in Table 3. a. Forest plot for SS vs SL+LL model, b. Forest plot for LL vs SL+SS model. (TIF) [file pone.0235512.s009.tif]

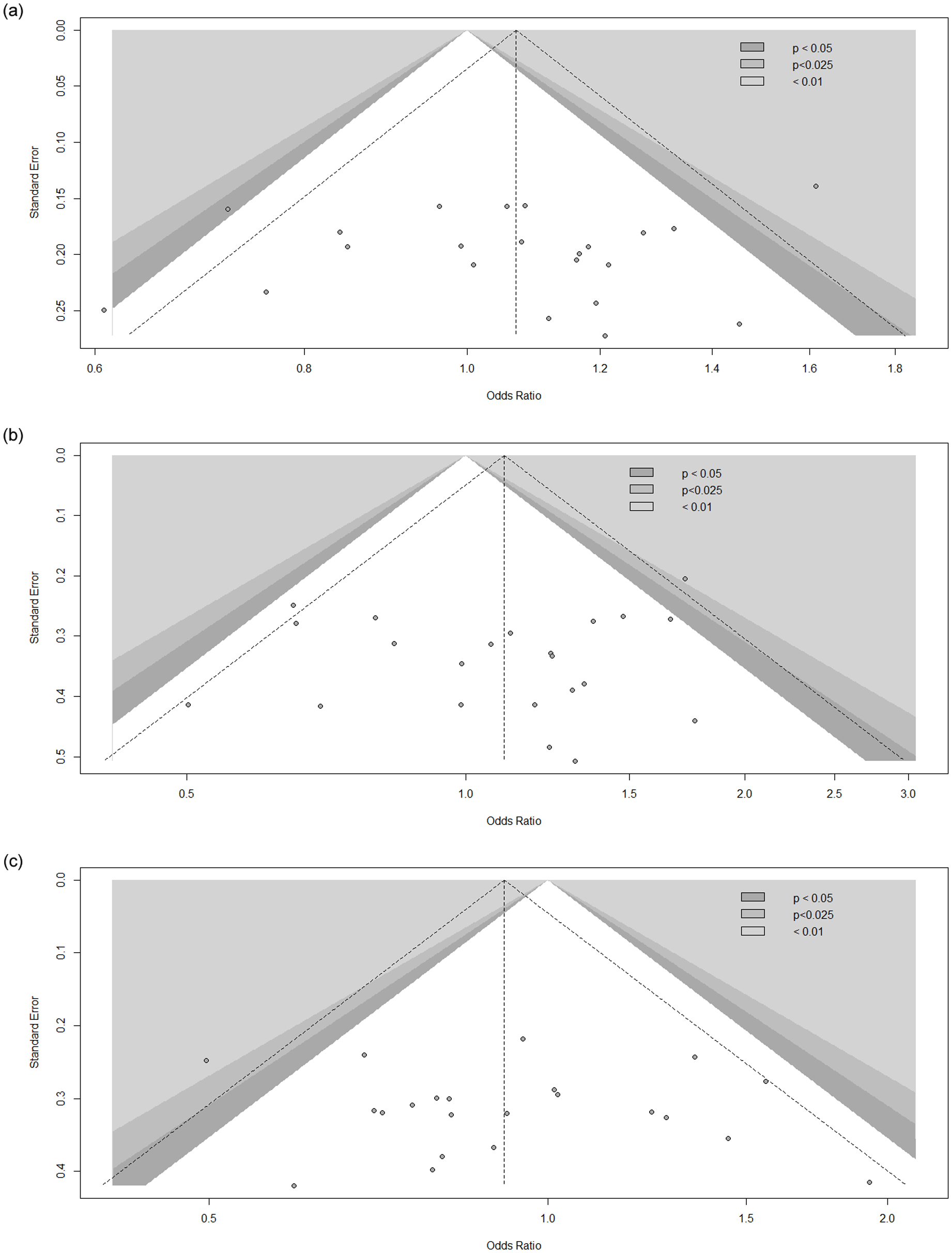

Supplement: S3 Fig — a. Funnel plot for S vs L model b. Funnel plot for SS vs SL+LL model, c. Funnel plot for LL vs SL+SS model. (TIF) [file pone.0235512.s010.tif]

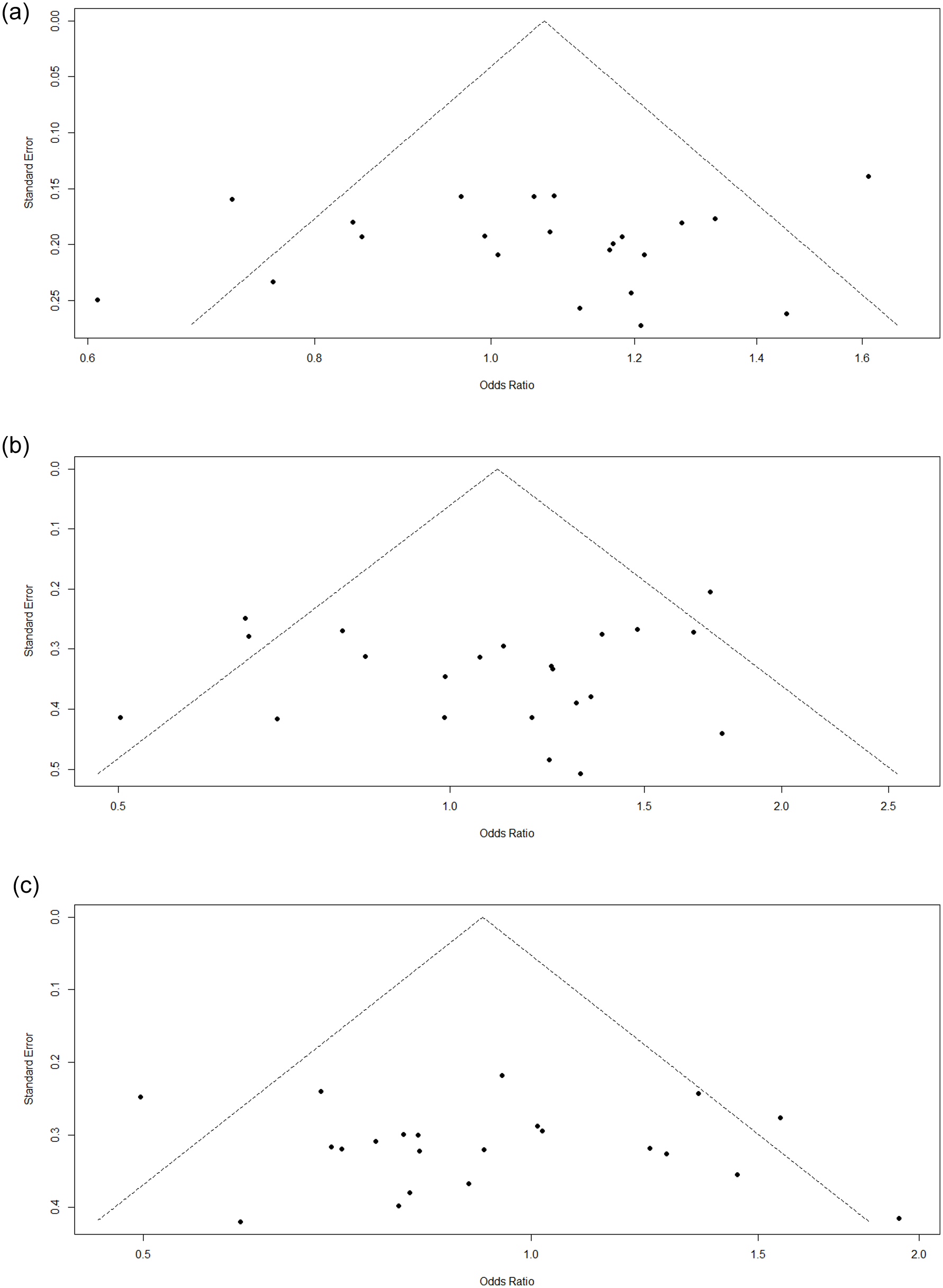

Supplement: S4 Fig — Funnel plots without missing studies a. for S vs L model, b. for SS vs SL+LL model and c. for LL vs SL+SS model. (TIF) [file pone.0235512.s011.tif]
