# Supplementary material for: The role of 5-HTTLPR in autism spectrum disorder: New evidence and a meta-analysis of this polymorphism in Latin American population with psychiatric disorders
Source: PLoS One. 2020 Jul 2;15(7):e0235512. doi: 10.1371/journal.pone.0235512 (PMC7332001; doi:10.1371/journal.pone.0235512)
Supplement: S1 Checklist — (DOC) [file pone.0235512.s012.doc]

| **Section/topic** | | **#** | | **Checklist item** | **Reported on page #** |
| --- | --- | --- | --- | --- | --- |
| **TITLE** | | | | |  |
| Title | | 1 | | The role of 5-HTTLPR in autism spectrum disorder: New evidence and a Meta-Analysis of this polymorphism in Latin American population with psychiatric disorders | Title section |
| **ABSTRACT** | | | | |  |
| Structured summary | | 2 | | The autism spectrum disorder (ASD) is a complex disorder encompassing a broad phenotypic and genotypic variability. The short (S)/long (L) 5-HTTLPR polymorphism has a functional role in the regulation of extracellular serotonin levels and both alleles have been associated to ASD. Most studies including European, American, and Asian populations have suggested an ethnical heterogeneity of this polymorphism; however, the short/long frequencies from Latin American population have been under-studied in recent meta-analysis. Here, we evaluated the 5-HTTLPR polymorphism in Colombian individuals with idiopathic ASD and reported a non-preferential S or L transmission and a non-association with ASD risk or symptom severity. Moreover, to recognize the allelic frequencies of an under-represented population we also recovered genetic studies from Latin American individuals and compared these frequencies with frequencies from other ethnicities. Results from meta-analysis suggest that short/long frequencies in Latin American are similar to Caucasian population but different to African and Asian regions. | Abstract section |
| **INTRODUCTION** | | | | |  |
| Rationale | | 3 | | Several studies evaluating the 5-HTTLPR polymorphism have suggested an ethnical heterogeneity. To further understand the ethnic component of this polymorphism a meta-analysis aiming to evaluate the heterogeneity of the S and L alleles in an under-represented and highly admixed population such as Latin America population was performed. | Introduction section, Paragraph 3 |
| Objectives | | 4 | | To recognize the allelic frequencies of 5-HTTLPR polymorphism in an under-represented population | Introduction section, Paragraph 3 |
| **METHODS** | | | | |  |
| Protocol and registration | | 5 | | Not applicable |  |
| Eligibility criteria | | 6 | | The inclusion criteria were (1) full text article in English or Spanish languages (2) case-control studies evaluating the polymorphism in psychiatric disorders (3) genotypic or allelic frequency provided (4) no deviation from Hardy-Weinberg Equilibrium (HWE) in controls. | “Literature search” of “Materials and Methods” section and “Supporting information”, S1 Fig |
| Information sources | | 7 | | We screened, without date restriction, published articles that evaluate the association of 5-HTTLPR and psychiatric disorders in PubMed, ScienceDirect and Scielo databases up to April 2020. | “Literature search” of “Materials and Methods” section and “Supporting information”, S1 Fig |
| Search | | 8 | | To minimize the chance of missing relevant studies the search terms in PubMed and ScienceDirect databases were “HTTLPR” “SLC6A4” AND the name of each country encompassed in South and Central America [each separated by the Boolean operator OR). For the Scielo database, we searched published literature in the indexed journals from Latin American countries | “Literature search” of “Materials and Methods” section and “Supporting information”, S1 Fig |
| Study selection | | 9 | | Included reports fulfilled the inclusion criteria: (1) full text article in English or Spanish languages (2) case-control studies evaluating the polymorphism in psychiatric disorders (3) genotypic or allelic frequency provided (4) no deviation from Hardy-Weinberg Equilibrium (HWE) in controls | “Literature search” of “Materials and Methods” section and “Supporting information”, S1 Fig |
| Data collection process | | 10 | | The data extraction was carried out by two investigators independently. Any disagreement was resolved though discussion. | “Literature search” of “Materials and Methods” section and “Supporting information”, S1 Fig |
| Data items | | 11 | | The collected data of each study was reference, country of origin, evaluated trait, number of individuals and allelic/genotypic frequencies. | “Literature search” of “Materials and Methods” section and “Supporting information”, S1 Fig |
| Risk of bias in individual studies | | 12 | | As inclusion criteria we confirmed that results were provided as a case-control analysis and the control individuals did not deviate from Hardy-Weinberg Equilibrium (HWE). We also carried out a sensitivity analysis removing each study for every meta-analysis. | “Literature search” and “statistical analysis”of “Materials and Methods” section and “Supporting information”, S1 Fig |
| Summary measures | | 13 | | The pooled Odds Ratio (OR) was evaluated under (S vs L), (SS vs LL+LS) and (LL vs SS+SL) models with fixed-effect or random-effect models according to I^2^ value (Fixed-effect for I^2^ <50% and Random-effect for I^2^ > 50%). The pooled odds ratio (OR) was expressed with the corresponding 95% confidence interval (CI). | “Statistical analysis” of “Materials and Methods” section |
| Synthesis of results | | 14 | | The heterogeneity across studies was estimated by the Cochran’s Q test and I^2^ statistic. The pooled OR was assessed in a fixed-effect model for I^2^ <50% and random-effect model for I^2^ > 50%. | “Statistical analysis” of “Materials and Methods” section |
| Risk of bias across studies | 15 | | The publication bias was assessed with funnel plots and quantitatively evaluated with Egger’s regression and Begg’s rank correlation. The trim and fill method was used to estimate potential missing studies. | | “Statistical analysis” of “Materials and Methods” section |
| Additional analyses | 16 | | -- | | -- |
| **RESULTS** | | | | |  |
| Study selection | 17 | | A total 112 reports were found in databases, fourteen duplicated reports were removed. These 98 were screened by title and abstract and 63 were excluded for not evaluating psychiatric disorders, no genotype for the 5-HTTLPR polymorphism was present or because it corresponded to reviews. From the 35 full text articles, 17 were excluded because they were family-based studies, studies evaluating cases and no controls, population without a clinical diagnosis and studies that did not evaluate the Latin American population or the association of 5-HTTLPR with psychiatric disorders. If overlapped samples were used in different studies, we kept the study with the largest sample size. No response was obtained from the authors that were contacted. After all the filters, 18 articles fulfilled the inclusion criteria. | | “Results” section, Paragraph 4 and “Supporting information”, S1 Fig |
| Study characteristics | 18 | | Thirteen case-control studies met the inclusion criteria in PubMed database: two from Colombia, three from Mexico, one from Argentina and seven from Brazil. In ScienceDirect database we found an additional study from Brazil and in Scielo database we found four additional studies: two from Colombia, one from Mexico and one from Brazil. | | “Results” section, Paragraph 4 and “Supporting information”, S1 Fig |
| Risk of bias within studies | 19 | | With sensitivity analysis the pooled ORs did not yield different conclusions before and after exclusion of the study. | | “Results” section, Paragraph 5 and “Supporting information”, S7 table. |
| Results of individual studies | 20 | | Results of individual studies are summarized in forest plots (see Fig 1 and S2 Fig) | | “Results” section, Fig 1, Table 3 and “Supporting information”, S2 Fig. |
| Synthesis of results | 21 | | The Latin American meta-analysis performed under three models (S vs L), (SS vs SL+LL) and (LL vs SL+SS) reflected a heterogeneity of 33.1%, 19.5% and 13.7%, respectively (see Table 3, Fig 1 and S2 Fig). Fixed-effect model was selected for quantitative analysis based on heterogeneity results. The pooled OR in either of the three models failed to find significant association (see Table 3) suggesting that the 5-HTTLPR polymorphism does not increase the risk for psychiatric disorders in Latin American population (see Fig 1 and S2 Fig). The recalculated OR with trim and fill method did not yield different conclusions (see Table 3) | | “Results” section, Paragraph 5, Table 3, Fig 1, and “Supporting information”, S2 and S4 Figs |
| Risk of bias across studies | 22 | | Funnel plots Egger´s and Begg’s test confirmed a not significant *p* value suggesting that results are not biased by studies (see table 3).  Trim and fill method did not identify missing studies for (S vs L), (SS vs LL+LS) and (LL vs SS+SL) models (see S4 Fig) and the recalculated OR did not yield different conclusions (see Table 3) just like the sensitivity analysis (S6 table). | | “Results” section, Paragraph 5, Table 3, and “Supporting information” S3 - S4 Figs and S6 table. |
| Additional analysis | 23 | | --- | | --- |
| **DISCUSSION** | | | | |  |
| Summary of evidence | 24 | | Although the S allele has been associated with increased risk for psychiatric disorders, our meta-analysis revealed no significant heterogeneity among studies, no publication bias and failed to find an association between 5-HTTLPR and a risk for psychiatric disorders. Comparing our frequencies with frequencies reported in other continents, the Latin American frequencies are more similar to those reported in Caucasian population, while S allele has been greater in Asian population and L allele greater in African population. | | “Discussion” section, Paragraph 5 |
| Limitations | 25 | | None of Latin American studies had a strict genetic control for population sub-structure between cases and controls and according to demographic history of these admixed countries substructure cannot be discarded. | | “Discussion” section, Paragraph 6 |
| Conclusions | 26 | | A meta-analysis evaluating this polymorphism in Latin American regions suggests that frequencies of short/long alleles in this under-represented population are relatively homogeneous to frequencies reported in Caucasian populations. | | “Discussion” section, Paragraph 6 |
| **FUNDING** | | | | |  |
| Funding | 27 | | There is no funding for this systematic review. | | None |

*From:*  Moher D, Liberati A, Tetzlaff J, Altman DG, The PRISMA Group (2009). Preferred Reporting Items for Systematic Reviews and Meta-Analyses: The PRISMA Statement. PLoS Med 6(6): e1000097. doi:10.1371/journal.pmed1000097

For more information, visit: **www.prisma-statement.org**.

Page 2 of 2
