# Supplementary material for: The role of 5-HTTLPR in autism spectrum disorder: New evidence and a meta-analysis of this polymorphism in Latin American population with psychiatric disorders
Source: PLoS One. 2020 Jul 2;15(7):e0235512. doi: 10.1371/journal.pone.0235512 (PMC7332001; doi:10.1371/journal.pone.0235512)
Supplement: Attachment — Submitted filename: Response reviewer.docx [file pone.0235512.s013.docx]

Response reviewer

Manuscript PONE-D-20-10849

Dear

**Giuseppe Novelli**
Academic Editor
PLOS ONE

We appreciate the careful reading and the provided feedback on the manuscript “The role of 5-HTTLPR polymorphism in Autism Spectrum Disorder and other psychiatric disorders in the Latin American population”. We changed the original title to “**The role of 5-HTTLPR in autism spectrum disorder: New evidence and a Meta-Analysis of this polymorphism in Latin American population with psychiatric disorders**”. We incorporated suggestions made by the reviewers and editor with track changes in the “Revised Manuscript with track changes” and answer them below:

1. Please ensure that your manuscript meets PLOS ONE's style requirements, including those for file naming.

A/ We adjusted the revised manuscript file according to PlosOne requirements.

We also included new tables (S1, S2 and S3 Tables) to fulfill publicly available information belonging to this manuscript. S1 Table includes genotypic information of trios and ADIR and ADOS scores for each individual with ASD. S2 Table has the genotypic information of controls. The S3 table includes the statistical results for each model.

2. PLOS requires an ORCID D for the corresponding author in Editorial Manager on papers submitted after December 6th, 2016. Please ensure that you have an ORCID iD and that it is validated in Editorial Manager.

A/ We included the ORCID ID of the corresponding author as follows https://orcid.org/0000-0003-2113-9266

Additionally, while reviewing the prisma checklist we encountered an article that contained all the genetic data but was initially excluded because it did not contain Hardy-Weinberg information. With the information provided by the authors in the article it was possible for us to obtain the HW results and therefore include it in our study. The results with the new information did not change the overall results

Reviewers' comments:

Reviewer's Responses to Questions

**Comments to the Author**


 Reviewer # 1:

The article, despite its simplicity, is interesting and well-focused in the descriptive part. It would be appropriate to adapt the Discussion section accordingly, avoiding elements of repetitiveness, in order to make the reading more smooth and pleasant.

According to the Preferred Reporting Items for Systematic Review and Meta-analysis statement (PRISMA checklist 2009) we suggest revising the title to introduce a statement regarding meta-analysis work.

A/ We appreciate your comments, they are very helpful and aimed to improve the quality of our work. _The original tittle “The role of 5-HTTLPR polymorphism in Autism Spectrum Disorder and other psychiatric disorders in the Latin American population” was modified to “**The role of 5-HTTLPR in autism spectrum disorder: New evidence and a Meta-Analysis of this polymorphism in Latin American population with psychiatric disorders**” to include the meta-analysis performed

It might be useful to describe the information sources including databases’ dates of coverage and eventual contact with study authors. Aiming for a stronger significance of the results, we suggest to include any assessment of publication bias and to describe the methods used to investigate the presence of missing studies. We also recommend specifying whether duplicates have been found. If so, the authors might want to insert the duplicates exclusion step in the flow chart (S1 Figure) in the screening section.

A/ We included the PRISMA checklist 2009 that contains information regarding dates, number of duplicated studies and authors contact in the paragraph of “Literature search”. Moreover, the paragraph “Statistical analysis” was modified to include the methodology used to evaluate for publication bias (Funnel plots, Egger’s / Begg’s tests) and the presence of missing studies (Trim and fill method).

A sensitivity assessment was also included to evaluate a risk of bias within selected studies.

The section Literature Search and Statistical Analysis was modified to include all these aspects.

In the text line 119, the information in the first parenthesis “(S vs )” seems to be incomplete.

The L letter was added to correct this mistake.

In the text lines 130-133, we recommend the substitution of the term “synapsis” (that is the pairing of homologous chromosomes during the first prophase of meiosis) with the term “synapse”. We also recommend to rephrase the sentence from text line 132 to 133.

We substituted the “Synapsis” by “Synapse” term. Moreover, we rephrased the next sentence for better understanding.

Reviewer #2:

I think this work is interesting and able to offer a more comprehensive point of view about the relation between ASD and 5-HTTLPR polymorphism, especially of course in Latin American population. All the tables presented are clear and easy to comprehend.

The principal Flaw I personally found in the article, was how the “Discussion” section was constructed. Some topics were actually proposed more than once, rather than being articulated cohesively. For example, while explaining the preferential transmission of L or S alleles: lines 140-144 vs lines 176-178 (in association with ethnicity). Some sentences are a little bit redundant and could have been better formulated, like: “Comparing our frequencies with… of the L allele” (lines: 194-198).

We appreciate your comments, they are very helpful and aimed to improve the quality of our work. We removed repetitive information previously consigned in these paragraphs and rephrased sentences to provide a better formulation of this information.

At line 119 there is a letter missing: (S vs L*).

The L letter was added to correct this mistake.
